# Speed Improvement in Image Stitching for Panoramic Dynamic Images during Minimally Invasive Surgery

**DOI:** 10.1155/2018/3654210

**Published:** 2018-12-04

**Authors:** Dinh Thai Kim, Van Thang Nguyen, Ching-Hwa Cheng, Don-Gey Liu, Kai Che Jack Liu, Kai Che Jack Huang

**Affiliations:** ^1^Program of Electrical and Communications Engineering, Feng Chia University, 40724 Taichung, Taiwan; ^2^University of Information and Communication Technology, Thai Nguyen, Vietnam; ^3^Department of Electronic Engineering, Feng Chia University, 40724 Taichung, Taiwan; ^4^IRCAD Taiwan/AITS, Changhua, Taiwan

## Abstract

Minimally invasive surgery (MIS) minimizes the surgical incisions that need to be made and hence reduces the physical trauma involved during the surgical process. The ultimate goal is to reduce postoperative pain and blood loss as well as to limit the scarring area and hence accelerate recovery. It is therefore of great interest to both the surgeon and the patient. However, a major problem with MIS is that the field of vision of the surgeon is very narrow. We had previously developed and tested an MIS panoramic endoscope (MISPE) that provides the surgeon with a broader field of view. However, one issue with the MISPE was its low rate of video stitching. Therefore, in this paper, we propose using the region of interest in combination with the downsizing technique to improve the image-stitching performance of the MISPE. Experimental results confirm that, by using the proposed method, the image size can be increased by more than 160%, with the image resolution also improving. For instance, we could achieve performance improvements of 10× (CPU) and 23× (GPU) as compared to that of the original method.

## 1. Introduction

In minimally invasive surgery (MIS), the surgeon uses a variety of techniques to operate while ensuring that the patient is subjected to as few incisions as possible. In general, MIS is safer than open surgery and allows the patient to recover faster while experiencing less pain and scarring. MIS is a highly successful modern surgical method and involves inserting the surgical instruments and an endoscopic camera into the patient's body through a small incision or a natural orifice. However, the limited field of view is the most challenging aspect of this surgical procedure. The narrow view of the endoscopic camera prevents the surgeon from being able to image the entire surgical field with clarity. This can make the procedure difficult and increase the uncertainty involved. Hence, MIS is especially difficult for less-experienced surgeons.

In the field of image processing, the process of combining multiple overlapping images into a larger image is known as mosaicing or image stitching. Image-mosaicing methods can be classified into two categories: the direct method and the features-based method [1]. In the case of the direct method, all the available image data are used instead of a set of sparse features extracted from the images. Hence, the method can provide very accurate registrations. However, the initial estimation parameters must lie close to the true solution, and there must be a high degree of overlap between the images for convergence. The features-based method, on the other hand, does not require an initialization process, and algorithms that can match distinctive image features, such as the scale invariant feature transform (SIFT) [2], speeded-up robust features (SURF) [3], and oriented FAST and rotated BRIEF (ORB) [4], are used for estimating the alignment parameters. Furthermore, the use of sparse features accelerates the estimation process and improves real-time performance. A comprehensive review of the literature on this method has been published by Szeliski [1].

Features-based image-stitching methods have been implemented for medical applications. For instance, Zanet et al. [5] proposed a method for the automatic mosaicing of the images from a retinal slit lamp using SURF. There have also been other studies on the mosaicing of retinal slit lamp images [6–8]. For example, Ji et al. [9] demonstrated the fusion of images of local prostate lesions using SURF.

In minimally invasive surgery, there have been some studies done as early as around 2009. Behrens et al. [10] demonstrated the mosaicing of endoscopic bladder images from a sequence video using SIFT. After that, they developed a multithreaded image-mosaicing algorithm using SURF to perform the mosaicing of bladder images in real time [11]. Iakovidis et al. [12] proposed a novel method for automatic image stitching for efficient visualization using conventional wireless capsule endoscopy videos based on SURF. Recently, Yang et al. [13] proposed a scene-adaptive features-based approach for the mosaicing of placental vasculature images obtained during computer-assisted fetoscopic procedures using SURF.

In the development of technologies of the image-guided surgeries, the issue of image registration is most concerned, especially in gastroscopic surgeries. Pioneers have tried to make the images of the target organs fixed on the display screen and expand the field of vision (FOV) of the endoscope. Liu et al. [14] has built up a panoramic view for gastroscopy. They utilized a tracking device according to the image from a single-camera gastroscope with a dual-cubic projection method to create both local and panoramic views at the same time. In 2016, Hu et al. [15] proposed a robust technique for image registration which was named as homographic patch feature transform for sequential gastroscopic images. Both techniques would make the gastroscopy easier to handle and safer in gastroscopic surgeries.

Thus, image mosaicing is gradually being applied in medical applications, and the feasibility and assistive nature of the technique with respect to clinical applications have been explored widely. However, image mosaicing is limited to the compositing of images with small fields of view, such as those of blood vessels and urethras. Moreover, in all the studies described above, the image sequences mosaiced were obtained from a single moving endoscopic camera. This can yield only panoramic static images that do not reflect the changes that may occur in the shape of the organs or blood vessels being imaged outside the field of vision (FOV). Therefore, it is difficult to apply the technique during laparoscopic surgery, wherein the position and shape of the internal organs change frequently.

To solve this problem, we had proposed an MIS panoramic endoscope (MISPE) [16, 17] that is based on the use of two endoscopic cameras. The MISPE provides the surgeon with panoramic images that show the surgical area in its entirety and reflect the changes in the FOV owing to the use of two cameras. In this MISPE system, we found the image quality and speed of stitching needed to be improved for practical applications. To improve the quality of the mosaiced images, in this study, we use the graph-cut technique [18] in order to prevent moving objects from appearing in the overlap area. Moreover, we also use the multiband blending method [19] to smoothen the stitching results. Finally, to improve the image-stitching performance of the MISPE, we propose using the region of interest (ROI) in combination with the downsizing technique.

The features-based image-mosaicing process consists of two stages: image registration and image compositing. We had previously [20] introduced two techniques (choosing a smaller area from the original images and using zoomed-in images) to speed up the image registration process. However, the selection of a smaller area can only be done using the naked eye. When the two endoscopic cameras move close to or away from the surgical area, the two small areas must be selected again. This is difficult to do during actual surgery. To solve this problem, in this study, we propose using the ROI to identify the two small areas automatically when the two cameras move. We then combine this approach with the downscaling technique to speed up the image registration process. Furthermore, this also accelerates the seam mask estimation step of the image-compositing stage.

The rest of the paper is organized as follows: Section 2 introduces the proposed features-based image-stitching algorithm, while Section 3 describes the technique for improving the panoramic video performance. The experimental results are described and discussed in Section 4, while Section 5 lists the conclusions along with the directions for future research.

## 2. Proposed Features-Based Image-Stitching Algorithm

**Figure 1:**
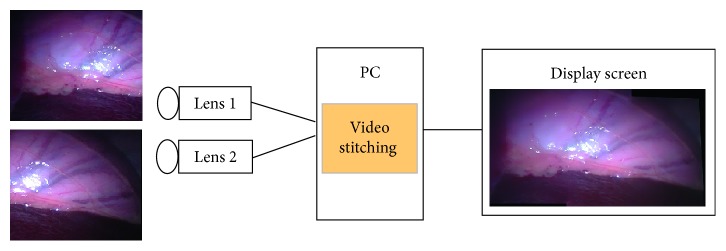
MISPE system.

To increase the viewing angle of the endoscope, we designed an MIS panoramic endoscope (MISPE) with two lenses at the tip of our device for primary investigation (Figure 1). In our MISPE, it consists of two lenses, which are connected to a PC via USB ports. In the video process, we developed a video-stitching module based on the features-based image-stitching algorithm to create a panoramic image, which is displayed on an image screen.

**Figure 2:**
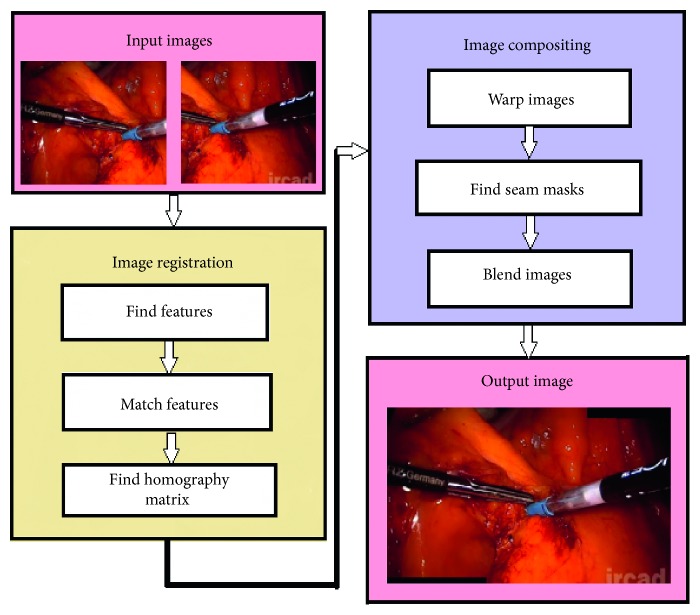
Flowchart of image-stitching process.

The features-based image-stitching algorithm comprises two stages: image registration and image compositing as illustrated in Figure 2.

The image registration stage has the following three steps: find the features, match these features, and then find the homography matrix. The purpose of these steps is to identify the coordinate relationship between the two source images. This stage is the most important one of the image-stitching process because it directly affects the correctness of the image-stitching results.

***Step 1*.**

Find features within images.

This step comprises two tasks: the first is to detect the feature points and the second is to construct descriptors of these points. Feature points are the characteristic points based on which an object can be recognized within an image. Because feature points only provide the positions of distinctive elements, matching them across different images requires characterizing them based on the extracted feature descriptors (feature vectors). A feature descriptor represents a subset of the total pixels in the neighborhood of the feature points. There are many algorithms available for searching for feature points and extracting their descriptors, such as SIFT [2], SURF [3], and ORB [4]. The feature point search algorithm used in this study is SURF [3], which allows for image scaling and rotation-invariant feature descriptions. Therefore, changing the viewing angle and size scale of the image within certain limits will not affect the correctness of the match results.

***Step 2*.**

Match features in different images.

The next step is to match the feature points in two different images (feature matching). Matching features is the process of defining the similarity between two features in two separate images based on the Euclidean distance (SSD) between the feature descriptors:

(1) $$\begin{array}{c} \text{SSD}=\sqrt{\overset{128}{\underset{i=1}{\sum }}{\left(x_{i}^{{\text{f}}_{1}}-x_{i}^{{\text{f}}_{2}}\right)}^{2}}, \end{array}$$

where (*x*_1_^f_1_^, *x*_2_^f_1_^,…, *x*_128_^f_1_^) and (*x*_1_^f_2_^, *x*_2_^f_2_^,…, *x*_128_^f_2_^) are the descriptors of the feature points in the two input images, respectively.

In this study, we used the nearest-neighbor-distance ratio [19], which is the ratio of the distances between the nearest and second-nearest neighbors. If the ratio is small, the nearest neighbor is a good match. We chose a ratio of 0.8, which eliminates 90% of the false matches while discarding less than 5% of the correct matches. This result was in consistence as reported in [2].

***Step 3*.**

Find homography matrix (H).

This step involves using the RANdom SAmple Consensus (RANSAC) algorithm [21] to remove the mismatched corresponding-point pairs and subsequently estimate the homography matrix based on the remaining set of corresponding pairs. The homography matrix is a 3 × 3 matrix with 8 degrees of freedom (DoF) as shown below:

(2) $$\begin{array}{c} \text{H}=\left[\begin{array}{ccc} {\text{h}}_{00} & {\text{h}}_{01} & {\text{h}}_{02}\\ {\text{h}}_{10} & {\text{h}}_{11} & {\text{h}}_{12}\\ {\text{h}}_{20} & {\text{h}}_{21} & 1 \end{array}\right], \end{array}$$

where h_*ij*_ represents the elements of the homography matrix.

After the image registration step is complete, the image-compositing stage yields wide-angle images. The image-compositing process also has three steps: warping the images, finding the seam masks for the warped images, and blending them.

***Step 4*.**

Warp images in same plane.

After the homography matrix has been determined during the image registration process, as described above, we use a perspective transformation to transform the two source images into two warped images in the same coordinate system such that they can be aligned to obtain a final composite image.

(3) $$\begin{array}{c} {\left(x,y\right)}_{\text{warp}}=\left(\frac{{\text{h}}_{00}x\;{+\text{ h}}_{01}y\;{+\text{ h}}_{02}}{{\text{h}}_{20}x\;{+\text{ h}}_{21}y+1}\text{,}\frac{{\text{h}}_{10}x\;{+\text{ h}}_{11}y\;{+\text{ h}}_{12}}{{\text{h}}_{20}x\;{+\text{ h}}_{21}y+1}\right), \end{array}$$

where (*x*, *y*) are the coordinates of a pixel in the source image, (*x*, *y*)_warp_ are the coordinates of a pixel in the warped image, and h_*ij*_ represents the elements of the homography matrix in equation (2).

***Step 5*.**

Find seam mask for warped images.

If the cameras are not aligned, they will have different views of the same scene, which will lead to the appearance of artifacts, such as those related to “misalignment” or “ghosting,” in the stitching results. This step is performed to find a seam that prevents the possibility of “ghosting” from the stitching results. In this study, we found the optimal seam for the images to be stitched using the graph-cut technique [18].

***Step 6*.**

Blend warped images.

After the images have been warped, a seam remains in output images owing to a difference in brightness between the two images. To solve this problem, we use a multiband blending method [19] to effectively smoothen out the stitching results.

## 3. Speed Improvement for Panoramic Video

As mentioned above, in this study, we propose a method for improving the video-stitching performance during MIS using two endoscopic cameras. Video stitching is the stitching of images in a frame-by-frame manner. In this paper, Frame 1 and Frame 2 are the labels used for the two images captured from Video 1 and Video 2, respectively.

**Figure 3:**
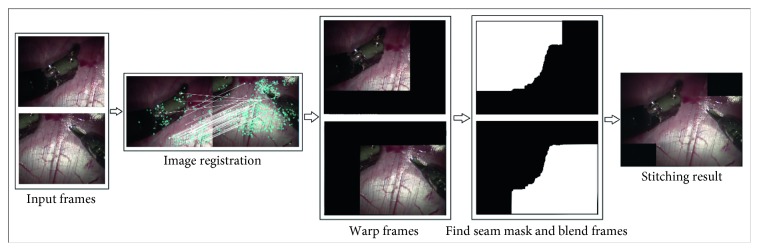
Conventional video-stitching algorithm.

The conventional video-stitching algorithm is as follows: the two frames are the two input images. During the image registration step, the algorithm searches for matching image features to determine the homography matrix. Next, the two input frames are transformed into two warped frames. The last step is to find a seam for the two warped frames and blend them.  Figure 3 illustrates these processes with the related image results.

**Figure 4:**
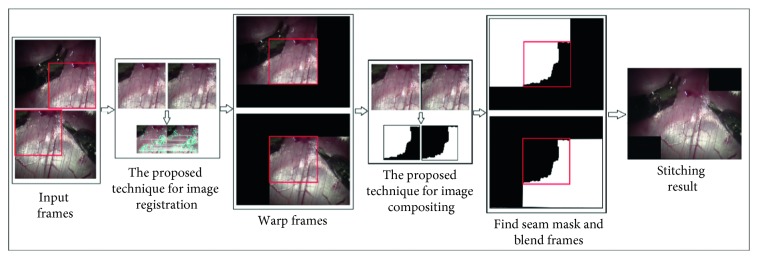
Proposed video-stitching algorithm.

An important parameter with respect to video stitching is the execution time of the algorithm. For practical applications, the video-stitching process needs to be a real-time one. Because the computational time for image processing is proportional to the size of the image being processed, large or high-resolution images would take a lot of time. Therefore, the proposed method aims to improve the video-mosaicing performance by improving both image registration and image compositing as demonstrated in Figure 4. The detail processes are described in subsections below.

### 3.1. Accelerating Image Registration

The purpose of image registration is to find corresponding point pairs between the two images and to subsequently determine the homography matrix and transform the two images on the same surface plane and align them. Because good matching pairs appear in the overlap region of the two images, using two small regions containing the overlap region for matching increases the accuracy and also speeds up the search for the corresponding point pairs of the original two images.

**Figure 5:**
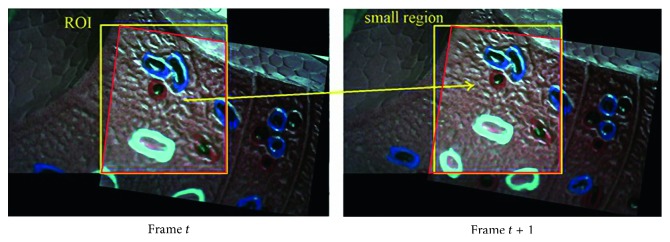
Overlap region during image stitching at frame *t* and frame *t* + 1 (red), ROI during image stitching at frame *t* (left, yellow), and small region during image stitching at frame *t* + 1 (right, yellow).

During laparoscopic surgery, the movement of the two endoscopic cameras is not too fast. Hence, the position and size of the overlap area of the two current frames and the two previous one are not very different. Furthermore, the previous frame's overlap is determined after the image-stitching process. Thus, the proposed technique uses the overlap region's size and the position of the two previous frames to determine the two small regions of the current frames for matching. This region is called the ROI.  Figure 5 depicts how we define the ROI at frame *t* and the small region at frame *t* + 1 during image stitching.

### 3.2. Define Region of Interest (ROI)

**Figure 6:**
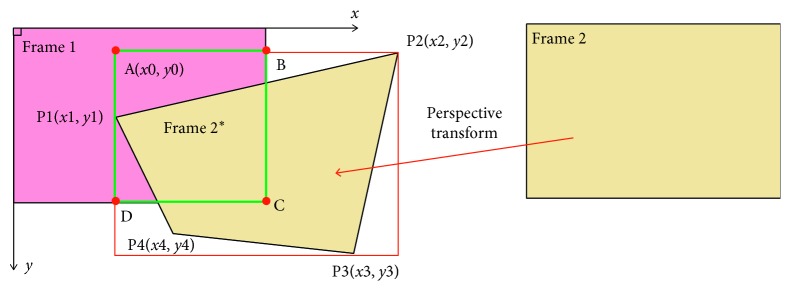
ROI of Frame 1. Four corners of Frame 2 are transformed into four points P1, P2, P3, and P4. Red rectangle is rectangle surrounding Frame 2^*∗*^'s edges and is parallel to Frame 1. The ROI of Frame 1 is intersection of Frame 1 within red rectangle (green rectangle).

After finding the homography matrix, we use a perspective transformation to transform Frame 2 into Frame 2^*∗*^ on the Frame 1 plane (Figure 6). Then, we define a rectangle such that Frame 2^*∗*^'s edges are parallel to Frame 1. The ROI of Frame 1 is the intersection of Frame 1 within this rectangle (the green rectangle ABCD). In the same manner, the ROI of Frame 2 is determined by transforming Frame 1 onto the same Frame 2 plane.

We assume that the four corners of Frame 2 after the transformation are *P*1(*x*1, *y*1), *P*2(*x*2, *y*2), *P*3(*x*3, *y*3), and *P*4(*x*4, *y*4). Hence, the ROI of Frame 1 is the region at position A(*x*0, *y*0) with a width $\bar{\text{AB}}$ and height $\bar{\text{AD}}$ in Frame 1. Their parameters are defined as follows:

(4) $$\begin{array}{c} \begin{array}{c} x0=\max\left(\min\left\{x1,x2,x3,x4\right\},0\right),\\ y0=\max\left(\min\left\{y1,y2,y3,y4\right\},0\right), \end{array} \end{array}$$

(5) $$\begin{array}{c} \bar{\text{AB}}=\min\left(\max\left\{x1,x2,x3,x4\right\},\text{width of Frame}1\right)-x0,\\ \bar{\text{AD}}=\min\left(\max\left\{y1,y2,y3,y4\right\},\text{height of Frame}1\right)-y0. \end{array}$$

The matching of the two ROIs can reduce the computational time if the two ROIs are reduced to a lower resolution (resized ROI region). For the downsizing of the images, the bilinear interpolation algorithm is used. The resized-scale value can be input manually to accelerate the image registration process. Because the homography matrix consists of nine elements (including the final element, which is equal to 1), one needs at least four correspondence points to determine the matrix. Hence, the scale value is selected so as to ensure that the number of good matches is not less than 4. In many of the experimental cases, we chose the scale value such that the original images could be resized to a resolution of 320 × 240.

On using the two resized ROI regions for the matching operation, the feature point's coordinates in the image change. Therefore, the homography matrix for the two original images must be changed as well. The algorithm for determining the homography matrix is as follows:

**Step 7 .**

Determine the two small regions from the current frames. The region is set to the whole frame for the first two frames. For the subsequent frames, the regions are determined based on the size and position of the ROI of the previous two frames.

***Step 2*.**

Resize the two small regions at a lower resolution (resized small region).

***Step 3*.**

Use the SURF algorithm to determine the homography matrix for the two resized small regions (H matrix), as described in the image registration step in Figure 2.

***Step 4*.**

Calculate the homography matrix for the two current frames.

(6) $$\begin{array}{c} {\text{H}}_{\text{homography}}=\left[\begin{array}{ccc} k & 0 & 0\\ 0 & k & 0\\ 0 & 0 & 1 \end{array}\right]\left[\begin{array}{ccc} 1 & 0 & x1\\ 0 & 1 & y1\\ 0 & 0 & 1 \end{array}\right]\text{H}\left[\begin{array}{ccc} 1 & 0 & -x2\\ 0 & 1 & -y2\\ 0 & 0 & 1 \end{array}\right]\left[\begin{array}{ccc} 1/k & 0 & 0\\ 0 & 1/k & 0\\ 0 & 0 & 1 \end{array}\right], \end{array}$$

Here, the homography matrix, H, is used to transform resized ROI 2 on the same plane as that of resized ROI 1. Further, (*x*1, *y*1) and (*x*2, *y*2) are the coordinates of the top-left corners of ROI 1 and ROI 2, respectively, in the coordinate system, while *k* is the resized-scale value.

### 3.3. Accelerating Image Composition

After the image registration process has been completed, we use the homography matrix to transform the two frames on the same plane (warped frames). The next step is to find the seam masks for these two warped frames. The aim is to determine the optimal boundary between the overlapping pixels of the two images in order to reduce the visual artifacts. In this study, we use the graph-cut algorithm [18] to determine the optimal seam between the two warped frames.

However, the graph-cut algorithm takes a lot of time. The computational time for the graph-cut algorithm from the OpenCV library for two images with average resolutions of 640 × 480 is more than 2 s for the CPU version and more than 1.5 s for the GPU version. Further, the computational time is proportional to the image size. In order to speed up the process, we propose using the ROI to determine the seam mask. Then, we resize the two ROIs to a lower resolution in order to reduce the computational time for estimating the seam mask. The details are presented below (Figure 4):

***Step 1*.**

Decide ROI 1 in warped-Frame 1 and ROI 2 in warped-Frame 2 using equations (4) and (5).

***Step 2*.**

Resize ROI 1 and ROI 2 to lower resolutions to find the seam mask (ROI-seam-masks).

***Step 3*.**

Resize these ROI-seam-masks to the original resolution.

***Step 4*.**

Then, copy these ROI-seam-masks to position (*x*0, *y*0) using equation (4) in order to obtain the two seam masks for the two warped frames.

## 4. Results and Discussion

The OpenCV and OpenCL programming languages are both used in the proposed technique. The program was executed using an Intel *i*3 CPU and a GTX750Ti Nvidia GPU with 8 GB RAM. The GPU plays a major role in the optimization of processing in PCs. The stitching performance can be accelerated by performing CPU and GPU operations simultaneously. The two endoscopic cameras used were of the same type (2.0 MP USB digital Microscope).

### 4.1. Video-Stitching Results

To validate the efficacy of the proposed algorithm, we performed image mosaicing on two videos using both a phantom model and in vivo animal experiments.

**Figure 7:**
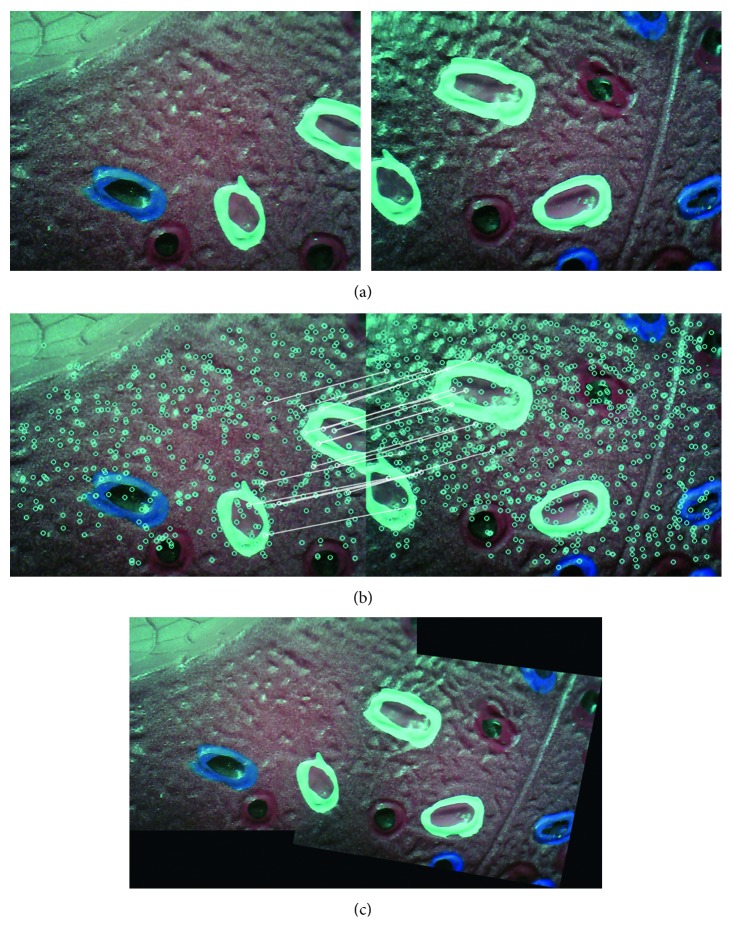
Type 1 detail in image-stitching result (phantom model). The result expands the original FOV of the input image by 60%. (a) Input images. (b) Match feature points. (c) Stitching result.

Figure 7(a) shows two input images captured using the two endoscopic cameras during the phantom experiment, while Figure 7(b) shows the matching features in the two input images, and Figure 7(c) shows the stitching result. Thus, the images confirm that the proposed method can expand the FOV of the original one by 160%.

**Figure 8:**
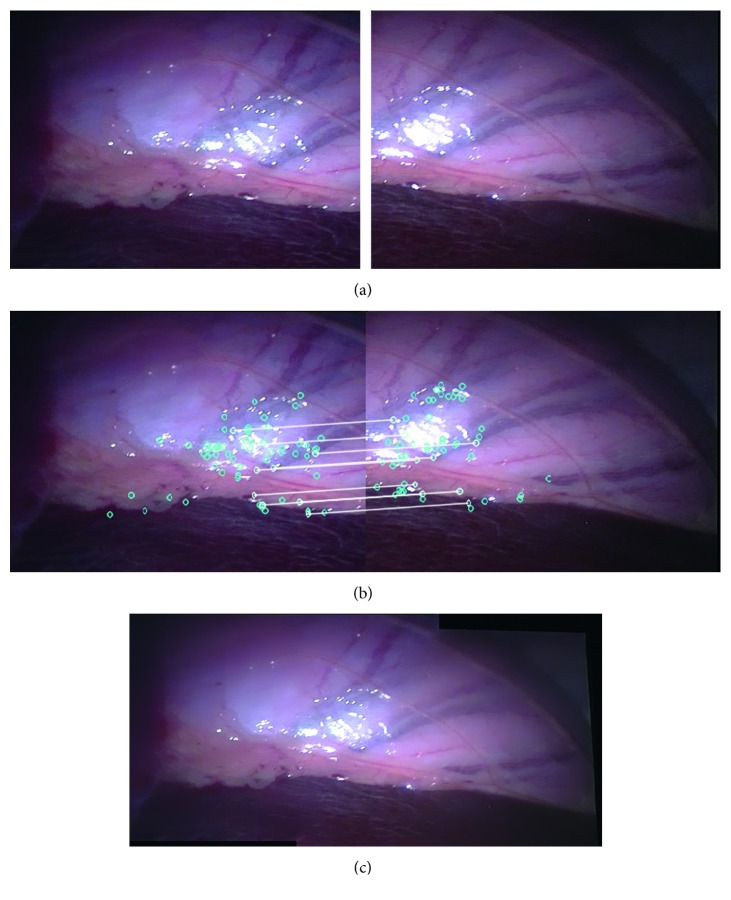
Type 2 detail in image-stitching result (in vivo animal experiment). The result expands the original FOV of the input image by 55%. (a) Input images. (b) Match feature points. (c) Stitching result.

Figure 8(a) shows two input images captured from two videos during an in vivo animal experiment, Figure 8(b) shows the matching features in the two input images, and Figure 8(c) shows the stitching result. Thus, it can be seen that the proposed method can expand the FOV of the original one by 155%.

### 4.2. Improvement in Video-Stitching Speed

The effectiveness of the proposed method was compared with that of the conventional one. The results of the comparison are described in this section. The stitching video was produced using the two endoscopic cameras at medium resolution (640 × 480). The program used for the performance comparisons was executed on both a CPU and a GPU.

#### 4.2.1. Image Registration Results

**Figure 9:**
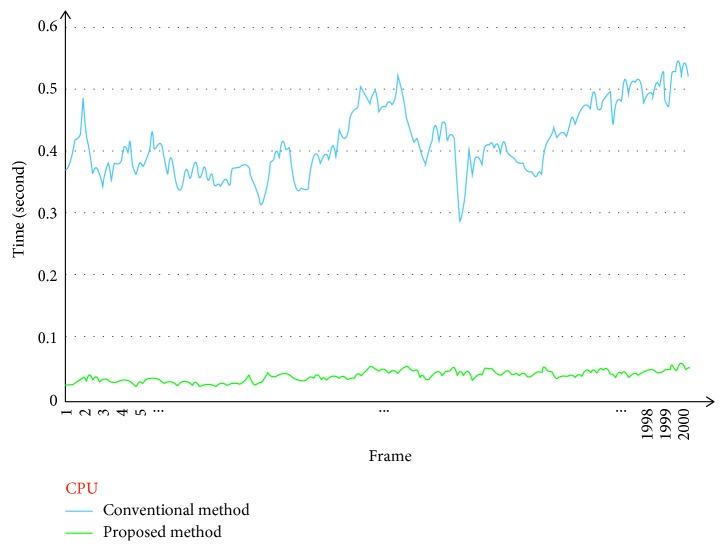
Comparison of image registration times for conventional method (blue) and proposed method (green) (CPU version).

**Figure 10:**
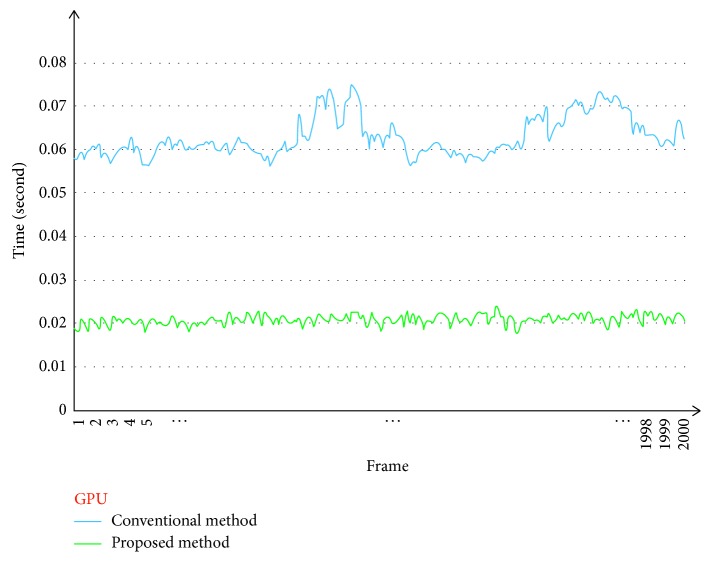
Comparison of image registration times for conventional method (blue) and proposed method (green) (GPU version).

Figures 9 and 10 show the computational times for the frame-by-frame image registration step performed using a CPU and GPU, respectively. For the videos with a resolution of 640 × 480, we chose a resized-scale value of 2 while ensuring that the number of good matches was sufficiently high. For larger resolution videos, the resized-scale value selected was higher, allowing for further improvements in performance.

Table 1 shows that the proposed method allowed for increases in the computation rate of 10.75 times (CPU) and 3.1 times (GPU) as compared to those for the conventional method.

#### 4.2.2. Seam Mask Estimation Result

**Figure 11:**
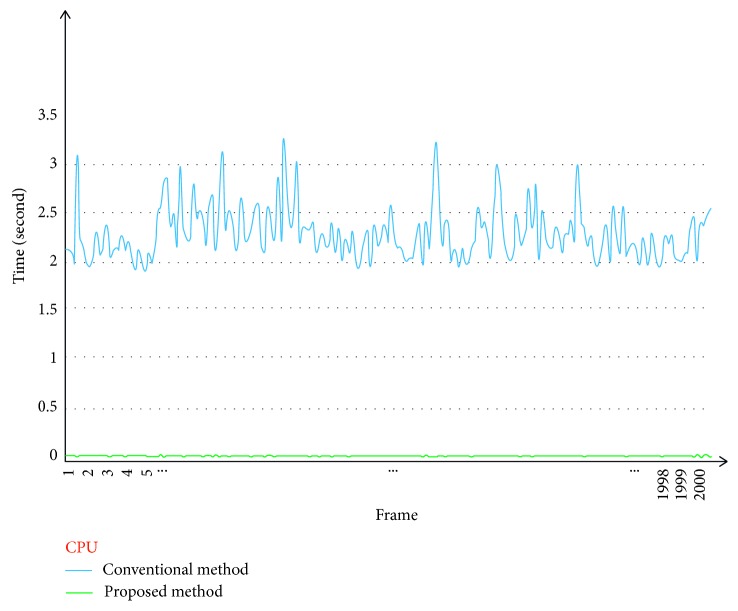
Comparison of seam mask estimation times for conventional method (blue) and proposed method (green) (CPU version).

**Figure 12:**
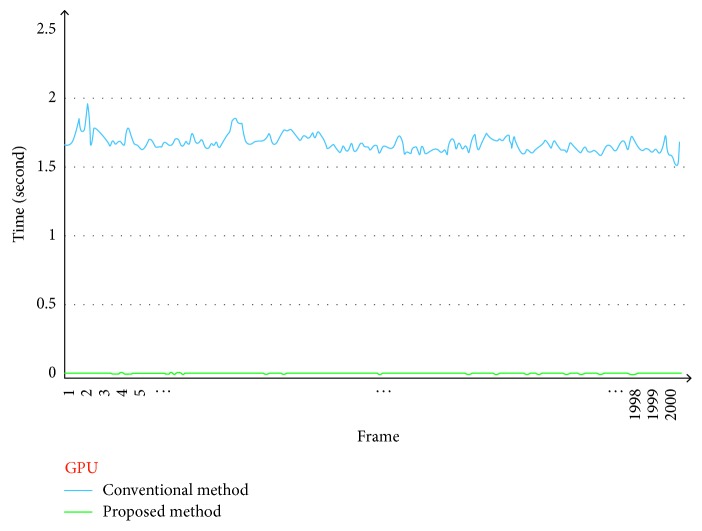
Comparison of seam mask estimation times for conventional method (blue) and proposed method (green) (GPU version).

Figures 11 and 12 show the computational times for seam mask estimation (frame by frame) as performed using a CPU and GPU, respectively. For the videos with a resolution of 640 × 480, we chose a resized-scale value of 10 while ensuring that the seam estimation quality was high. For larger resolution videos, the resized-scale value will be higher, resulting in additional improvements in performance.

As can be seen from Table 2, the proposed method increased the speed for finding the seam mask by 153 times (CPU) and 140 times (GPU) in comparison to those for the conventional method.

### 4.3. Discussion

The image-stitching process includes image registration and image compositing. Further, the image-compositing stage itself has three steps: the warping of the images, finding the seam masks, and the blending of the images. From the above-described results, we can calculate the total time for the image-stitching process.

Table 3 shows the average times for the video-stitching process without improvements (i.e., for the conventional method) and with improvements (i.e., for the proposed method) for 2000 consecutive frames. Thus, it can be seen that the proposed method results in improvements in both image registration and image compositing.

It can be seen that the conventional method takes 2.97 s on the CPU and 1.838 s on the GPU, which means that the frame rate is approximately 0.34 fps on the CPU and 0.54 fps on the GPU. Thus, the conventional method is slow both in the case of the CPU and the GPU for the image-stitching operation.

Table 3 shows the results for the proposed method as well, which has a frame rate of 3.33 fps on the CPU and 12.82 fps on the GPU. This means that that the proposed method results in an improvement of 10× on the CPU and 23× on the GPU as compared to those of the conventional method.

**Figure 13:**
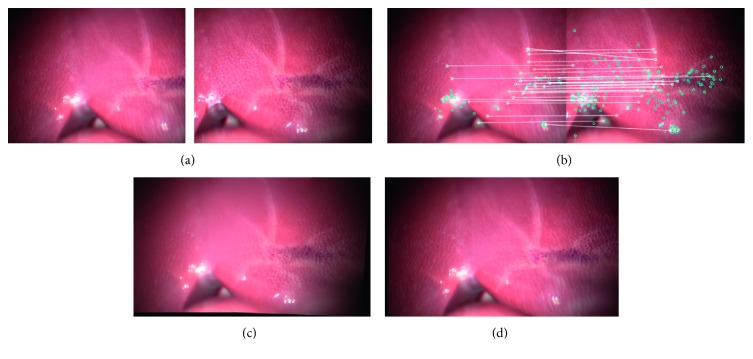
Image-stitching result for conventional method: (a) input images, (b) matching feature points, (c) stitching result, and (d) ground truth.

**Figure 14:**
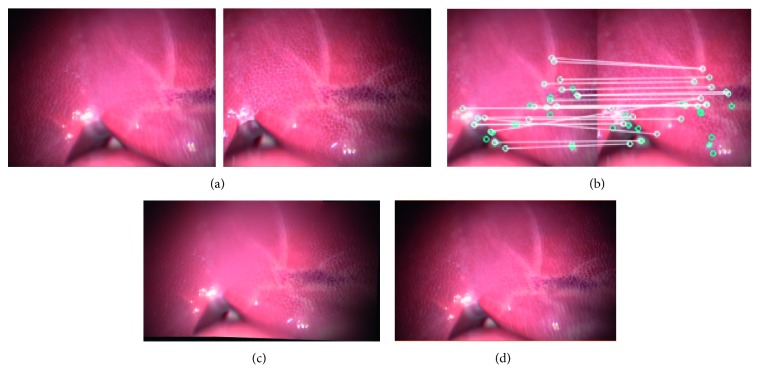
Image-stitching result for proposed method: (a) input images, (b) matching feature points, (c) stitching result, and (d) ground truth.

Figures 13 and 14 show the stitching results for the conventional and proposed methods. The images in (a) are the input images, while those in (b) show the results of feature matching. The image in (c) shows the stitching result while that in (d) shows the ground truth. It can be seen that the stitching results obtained using the proposed method are as natural as the ground truth. Furthermore, the stitching speed by our proposed method is higher while image quality is not degraded (Figure 14).

However, the proposed method has two limitations. The first is that, when the two endoscopic cameras move fast, the overlap between the two previous frames and the two current ones will vary widely in terms of location and size. Hence, the proposed method cannot be applied when using the ROI coordinates of the previous frames to estimate the small areas of the current frames. However, this is less likely to occur during laparoscopic surgery, as fast movements blur the image captured by the camera. The allowable camera velocity depends on the camera sensor and was found to be approximately 2.5 cm/s in this study. The second is that the image registration of the two current frames can fail owing to poor image quality or because the overlap region is missing. This will lead to an inaccurate or missing ROI. Hence, the image registration of the subsequent frames will also fail.

**Figure 15:**
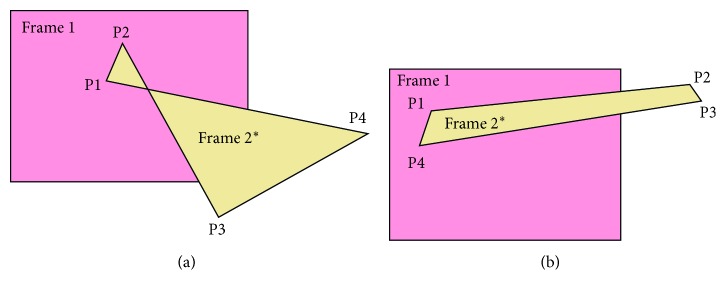
Transformation of Frame 2 into Frame 2^*∗*^: (a) nonquadrilateral and (b) quadrilateral.

To identify the cases where image registration fails, we determine the number and quality of the matching pairs between the two frames. If this number is less than 4, the number of equations available will not be enough to determine the homography matrix. On the other hand, even when the number is greater than 4, there are very few exactly matched pairs, and most of them are inaccurate; in this case even the homography matrix can be obtained, and Frame 2 will be transformed into a nonquadrilateral or an irregular shape as shown in Figures 15(a) and 15(b). Hence, the stitching results will be distorted and not meaningful to the surgeon. When these situations are encountered, the current frame's ROI is set to the whole frame and the following image-compositing stage would be skipped. The stitching process will be resumed on next frames.

**Figure 16:**
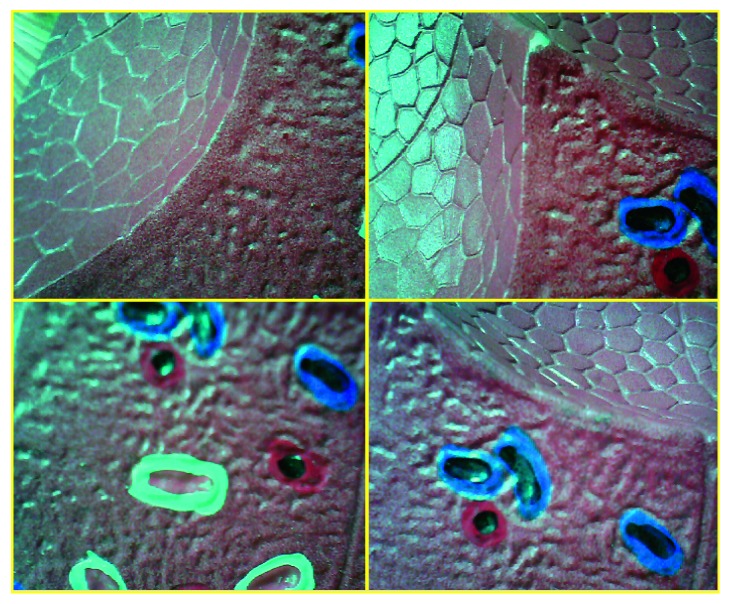
Images captured by four cameras.

**Figure 17:**
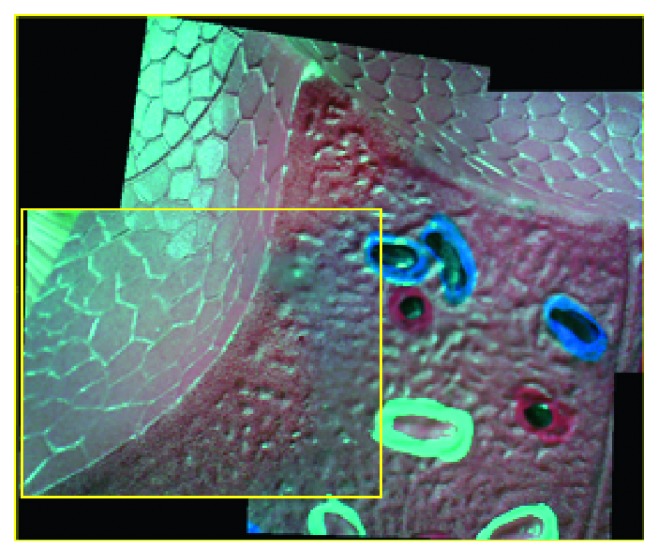
Result of image stitching of four input images (area expansion ratio is 300%).

In this study, we aimed to perform the real-time stitching of images from multiple endoscopic cameras. In Figures 16 and 17, the stitching results for four cameras are arranged in two rows.

The images indicate that the camera's viewing angle can be extended by approximately 300%. However, in the present study, we only focused on stitching the images captured by two endoscopic cameras owing to the real-time operational requirements of MIS.

## 5. Conclusions

In this study, we proposed an MISPE with a broader field of view. We have proposed a downsized ROI technique that can combine with SURF to improve the speed of the registration. In addition, we also combined the downsized ROI with graph-cut algorithm to speed up the image composition. The experimental results obtained showed that the MISPE can enhance the image size by up to 160%. As compared to the conventional method, the proposed one results in performance improvements of 10× (CPU) and 23× (GPU). The proposed technique can also be used for stitching video from a single camera or multiple ones. The technique was confirmed to be effective both with large-sized images and high-resolution ones. The frame rate for the video stitched from two endoscopic cameras at a resolution of 640 × 480 was determined to be 12.82 fps. In the future, we plan to further improve the performance of the real-time stitching operation when using multiple endoscopic cameras.

## Figures and Tables

**Table 1:** Average times for image registration step after 2000 frames.

Time (s)	CPU (s)	GPU (s)
Conventional method	0.430	0.062
Proposed method	0.040	0.020

**Table 2:** Average times for image seam mask estimation step after 2000 frames.

Time (s)	CPU (s)	GPU (s)
Conventional method	2.295	1.689
Proposed method	0.015	0.012

**Table 3:** Computational times for image stitching with/without improvements.

Method	CPU (s)	GPU (s)
Conventional method	2.970	1.838
Proposed method	0.300	0.078

## Data Availability

The data used to support the findings of this study are available from the corresponding author upon request.
