# Corrigendum to “Speed Improvement in Image Stitching for Panoramic Dynamic Images during Minimally Invasive Surgery”

**DOI:** 10.1155/2020/7810583

**Published:** 2020-09-17

**Authors:** Dinh Thai Kim, Van Thang Nguyen, Ching-Hwa Cheng, Don-Gey Liu, Kai Che Jack Liu, Shih Wei Wayne Huang

**Affiliations:** ^1^Program of Electrical and Communications Engineering, Feng Chia University, 40724 Taichung, Taiwan; ^2^University of Information and Communication Technology, Thai Nguyen, Vietnam; ^3^Department of Electronic Engineering, Feng Chia University, 40724 Taichung, Taiwan; ^4^IRCAD Taiwan/AITS, Changhua, Taiwan

In the article titled “Speed Improvement in Image Stitching for Panoramic Dynamic Images during Minimally Invasive Surgery” [1], the name of the last author was given incorrectly as Kai Che Jack Huang. The name should have been written as Shih Wei Wayne Huang, and the revised authors' list is shown above.
